# Etymologia: Pegivirus

**DOI:** 10.3201/eid2602.ET2602

**Published:** 2020-02

**Authors:** Ronnie Henry

**Keywords:** pegivirus, viruses, GB virus, hepatitis G virus, hepatitis

## Pegivirus [pegʺi-viʹrǝs]

In 1967, researchers studying non-A, non-B hepatitis identified a transmissible agent in the serum of a surgeon (initials G.B.) with acute hepatitis and named it the GB agent. In the 1990s, researchers from Abbott Laboratories identified 3 GB viruses (A, B, and C) at the same time as a group at Genelabs isolated RNA from patients with non-A, non-B hepatitis and named it hepatitis G virus. Later research showed that GB virus C and hepatitis G virus were the same species.

Subsequent phylogenetic analysis showed that GB viruses A and C (and GB virus D, later identified in bats) should be classified under a new genus, *Pegivirus* (because they cause persistent infection and because of the historic association with hepatitis G), and GB virus B should be classified as a second species (with hepatitis C virus) in the genus *Hepacivirus*. As of 2016, 11 species of *Pegivirus* had been identified (*Pegivirus A–K*).
